# Prevalence and molecular analysis of *Toxocara cati* in Baghdad Province

**DOI:** 10.5455/javar.2024.k788

**Published:** 2024-06-09

**Authors:** Zaid Khalid Alani, May Hameed Kawan

**Affiliations:** 1 College of Health Medical Techniques, Al-Bayan University, Baghdad, Iraq; 2 College of Veterinary Medicine, University of Baghdad, Baghdad, Iraq

**Keywords:** *Toxocara cati*, sequences, molecular, ITS2, zoonotic parasite

## Abstract

**Objective:**

This study aimed to detect *Toxocara cati* in cats by microscopic and molecular analysis using PCR, sequencing, and phylogenetic analysis.

**Materials and Methods:**

Randomly selected 200 cat feces samples were taken from various private veterinarian clinics in Baghdad. To identify eggs of *T. cati* by the flotation method, DNA from 100 cat feces was extracted, and one pair of ITS2 region-specific primers was used for polymerase chain reaction, followed by sequencing.

**Results:**

*Toxocara cati* infection rate was found to be 23 out of 100 fecal samples using PCR. Ten DNA product sequence data studies showed 98%–100% similarity to the 5.8S ribosomal RNA gene sequences found in the Gene Bank. The study incidence showed that the overall infection rate by microscopic examination was 23%, with no significant difference between stray cats (27%), and domestic cats (19%). After studying the effect of several epidemiological parameters on the infection rate, it was found that the infection rates of stray and domestic cats were higher in kittens under six months of age, at 46.1% and 27%, respectively, whereas rates were lower for the adult than six months was 11.5% of domestic cats and 14.7% of stray cats. The percentage of stray and domestic male cats that were registered was 35.5%, whereas the female cats registered were 20.6% and 17.5%, respectively.

**Conclusion:**

Cats are significant clinical reservoirs for zoonotic parasites. In Iraq, Baghdad has a high incidence of *T. cati* detections. Compared to conventional methods, PCR is thought to be a more sensitive, accurate diagnostic procedure that confirms the species’ identity.

## Introduction

The worldwide helminth parasite *Toxocara* spp. causes a variety of domestic, wild, and pet animals as final and paratenic hosts via a variety of ways of transmission, producing tough eggs that can withstand environmental conditions and long-lived larvae that inhabit tissues [1]. Pet animal intestines frequently contain the intestinal parasite *Toxocara* spp., especially in puppies and kittens, who are most vulnerable to parasitic diseases. These parasites’ ability to be spread by zoonotic agents increases the burden of disease and poses implications for public health. In fragile, impoverished communities in tropical and subtropical regions, where these animals roam freely, coexist with people, and are not dewormed, high prevalence rates have been reported [2]. Toxocariasis remains problematic worldwide as it causes systemic zoonotic infection in paratenic hosts like ruminants, persons, rabbits, poultry, and rodents [3]. The life cycle of this parasite occurs with the consumption of infectious eggs, which can be found in contaminated soil or the stools of infected cats. These eggs can hatch into adult worms that can reach a length of 10 cm, and the females can lay hundreds of thousands of eggs every day [4].

*Toxocara cati* is commonly detected in the gut of domestic cats worldwide, with differences in prevalence depending on geographical areas, laboratory procedures employed for diagnoses, and the structure of the tested feline population [5]. The ITS1 and ITS2 sections of rDNA can be used as genetic markers using polymerase chain reaction techniques for molecular systematic research of various parasite groups, and several studies used a PCR-based molecular technique based on the ITS sequence to differentiate *Toxocara* spp. eggs [6]. Infectious larvae can be generated by two capacities to release proteins (MUC-120) that support them to pass through the intestinal wall and then enter the intestinal tract and develop many tissues, including the lungs and liver [7]. The gastrointestinal helminths in the *Ascaridia* group can cause some clinical signs and make the eggs an environmental factor [8]. Contaminated water and food could be transferred by zoonotic agents, and there are extensive minor linkages with animal secretions [9]. In Iraq, the enzyme-linked immunosorbent assay was used as an accurate technique for the serodiagnosis of a large number of parasites in humans and animals [10]. However, in this study, *T. cati* was examined for the first time in Baghdad using DNA analysis, and a phylogenetic analysis was subsequently conducted.

## Materials and Methods

### Animals and sample collection

Two hundred fecal samples of cats were collected randomly from several private veterinary clinics in Baghdad. The samples were placed in plastic bags and refrigerated at 4°C until the processing was finished in a day. Using NaCl flotation techniques, *Toxocara* spp. eggs were examined under a microscope [11,12].

### Flotation using saturation with a NaCl solution

A 50-ml saturated sodium chloride solution was utilized, along with one to two gm of fecal material. The liquid was filtered and divided into two test tubes after ten minutes, with a coverslip applied to the surface of every test tube. The coverslips were laced on the glass slides and observed under the microscope at 10× [10].

### DNA extraction and PCR amplification

Geneaid Biotech Ltd. designed the Presto™ Stool DNA extraction kit, which was used to isolate DNA from 100 cat feces. The DNA extraction produced nucleic acids whose concentrations ranged from 82.2 to 210.3 ng/µl. One pair of oligonucleotide primers specific for the ITS2 region (5.8S ribosomal RNA gene) of *T. cati *was used. The sequences of these primers were the forward (5’-TGG TGC ATT CTT TCG CAA CG-3’) and the reverse (5’-GCC GAT GAC GTT ACC TCC AA-3’) to amplify a region of 232-bp. A thermocycler (Techne/UK) was programmed to perform the PCR. The following circumstances were met when performing amplifications: 30 cycles of initial denaturation at 94°C for 30 sec, annealing at 58°C for 30 sec, polymerase extension at 72°C for 30 sec, and a final cycle at 72°C for 10 min were performed. Then, 10 µl of each amplified sample mixed with loading buffer was loaded into the wells made in the 1.5% agarose gel, to which 3 µl/100 ml of ethidium bromide was added. The electrophoresis was run at 80 V for 1 h, and a DNA size marker was also loaded [4]. Products were visualized using a UV transilluminator (Vilber Lourmal/EEC) and photographed with a digital camera; positive results were determined when the sample DNA band had a size approximately similar to the desired product size. PCR water (4.5 µl), master mix (12.5 μl), forward primer (1.5 µl), reverse primer (1.5 µl), and 5 µl of template DNA were added to a 25 μl total volume mix for the PCR.

### Statistical analysis

The experiment’s data were statistically analyzed using the Statistical Analysis System (SAS, version 20.1). The study employed a two-way analysis of variance and the least significant differences post hoc test to evaluate any significant differences between the group means. The findings were presented as mean ± standard errors, with statistical significance defined as *p *< 0.05 [13].

## Results

### Detection of Toxocara spp. using a microscopic technique

**Figure 1.**
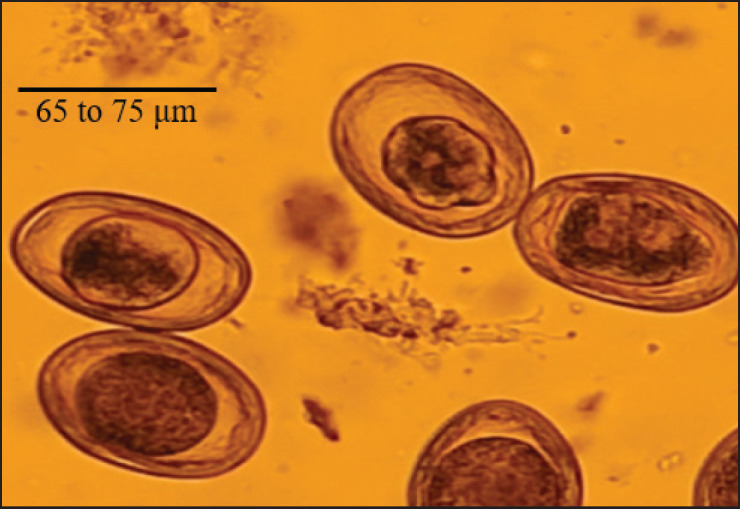
*Toxocara* spp. undeveloped eggs (40x).

Under 40× magnification, the eggs appeared round to ovoid, with a thick pitted wall and a total mean size ranging from 65 to 75 μm (Fig. 1). The total rate of infection in domestic and stray cats was 23% (46/200), with a non-significant difference (Table 1). Regarding the sex of the animals and age groups, the results of the investigation showed that there was no significant difference between domestic and stray cats (Tables 2 and 3). According to the survey study, the infection rate of *Toxocara* spp. during the months. The highest rate of infection was 66.6% (6/9) in stray cats, and in domestic cats, it was 71.4% (5/7) during July 2022. However, June 2022, February 2023, and April 2023 did not report any infections in domestic cats compared with November 2022, January 2023, and April 2023 in stray cats, where no infections were observed.

**Table 1.** Total rate of *Toxocara *spp. infection in domestic and stray cats by microscopic examination.

Percentage (%)	Positive samples	Samples examined	Animals
19.00	19	100	Domestic cats
27.00	27	100	Stray cats
23	46	200	Total
1.391 NS (0.238)	---	---	Chi-Square (χ^2^) (*p *value)

NS: non-significant.

**Table 2.** Prevalence of *Toxocara *spp*.* in domestic and stray cats according to the age groups.

Ages	Stray cats			Domestic cats		
	Samples examined	Positive samples	%	Samples examined	Positive samples	%
Kitten (<6 months)	39	18	46.1	48	13	27
Adults (>6 months)	61	9	14.7	52	6	11.5
Total	100	27	27	100	19	19

*Chi*-Square χ^2^ 0.016 *p* value 0.901

**Table 3.** Prevalence of *Toxocara* spp*.* in domestic and stray cats according the sex.

Sexes	Stray cats			Domestic cats		
	No. of samples examined	No. of positive samples	%	No. of samples examined	No. of positive samples	%
Male	42	15	35.7	57	10	17.5
Female	58	12	20.6	43	9	20.9
Total	100	27	27	100	19	19

*Chi*-Square (χ^2^) 0.038 *p* value 0.845

### Detection of T. cati using the PCR technique

**Figure 2.**
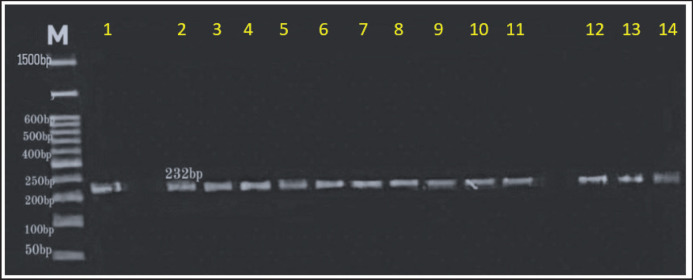
Agarose gel electrophoresis image shows the PCR product analysis of 232-bp of the conserved region of the 5.8S ribosomal RNA gene of *T. cati* from cat feces samples. Lanes 1 to 14 positive.

The results of DNA amplification by PCR showed that 23% (23/100) of the tested samples were positive. The PCR products were as expected, roughly 232 bp in length (Fig. 2). PCR was employed to examine samples taken from fecal cats randomly. All DNA samples were exposed to conventional PCR procedures for the recognition of *T. cati*. Using purified *T. cati* egg DNA (1.7 to 1.9), a conventional PCR was conducted using one pair of unique oligonucleotide primers that were freshly created for each ITS2 region of *T. cati*. The NanoDrop spectrophotometer was utilized for this process.

### DNA sequencing and phylogenetic tree analysis

**Figure 3.**
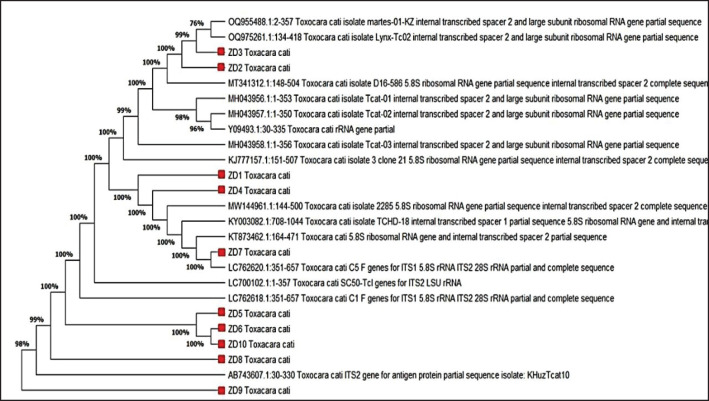
Dendrogram obtained for *T. cati* isolates/genotypes based on the ITS2 region of the 5.8S ribosomal RNA gene sequence by the maximum likelihood analysis at showing the phylogenetic tree of Iraq isolates with related Global strains.

In this study, 10 PCR products of the partially amplified ribosomal DNA gene of *T. cati* ITS-2 region were submitted for sequencing by Bioneer Company Korea. Applied Biosystems 3730xl DNA Analyzer, bidirectional Sanger sequencing was accomplished (Applied Biosystems, Foster City, CA) (Table 4). All of the sequences obtained were submitted to the National Center for Biotechnology Information (NCBI) for registration. The basic Local Alignment Search Tool was used to compare these sequences with other comparable sequences that had been uploaded to the GenBank database. Finally, the phylogenetic tree was constructed to include *T. cati* isolates from Iraq with other isolates obtained from other countries based on the above gene sequence (Fig. 3).

**Table 4.** Homology percentages between the *T. cati* of the local isolates compared with related sequences provided by NCBI.

*T. cati* Isolate No.	GenBank accession numbers	Identity %	International GenBank accession numbers	Country
T1	OR619698.1	100	MW144961.1	France
2	OR619699.1	100	MW144961.1	France
3	OR619700.1	99	KJ777157.1	India
4	OR619699.1	100	KY003082.1	Chine
5	OR619702.1	100	KT873462.1	Kyrgyzstan
6	OR619703.1	100	LC762620.1	Germany
7	OR619704.1	100	LC700102.1	Iran
8	OR619705.1	99	LC762618.1	Germany
9	OR619706.1	98	MH043956.1	Turkey
10	OR619707.1	100	LC762618.1	Germany

## Discussion

The incidence of intestinal helminths in cats, both stray and domestic, by microscopic examination was 23% (46/200). The results are consistent with another study, which recorded an infection rate of 25% with *T. cati* in Brazil [14]. The current study’s total infection rate was higher than what was previously documented in Mexico, which recorded 42.5% by flotation technique, and it was reported that 62.5% of Turkish have *T. cati*. [15,16]. In Estonia, 48.2% of cats were infected with *T. cati*; this rate was higher than the studies conducted in the neighboring country, Iran, where 42.6% were infected [17,18].

In Russia, 16.7% of cats were infected with *T. cati*, whereas other studies found that 52% of cats were infected [19,20]. The result of this study is lower than that in Mosul; it was 40% [21]. The percentage of infection was 12.9% in Baghdad by direct and flotation methods [9]. The prevalence rate of *T. cati* in this study was 23%, which disagrees with another study that recorded a 39.58% rate of infection with *T. cati* in fresh fecal specimens by flotation methods in Kirkuk City [22]. Last year in Sulaimani city, a total prevalence of 28.2% (22/78) with *T. cati *was reported by direct fecal smear and fecal floatation procedures [12]. The parasite may reactivate during pregnancy and pass through the placenta or breastfeed the fetus before or after delivery, which can affect the prevalence of the parasite [23]. Based on the age groups, kittens under six months of age recorded 46.1% (18/39) and 27% (13/48) rates of infection in stray and domestic cats, respectively. This result is higher than the study in Western Australia, which recorded the highest parasite prevalence of 34.3% in kittens [24]. An additional study in Portugal showed that the prevalence rate was comparable in kittens and adult cats, where the infection rate was 11.8% [25].

The overall prevalence rate of *T. cati* according to age groups was lower than a study conducted in Iran, which registered a 30% rate in cats over two years of age [26]. Regarding the sex of the animal, the present study revealed that there was no significant difference between stray and domestic cats, sex seemed to not affect the prevalence of the parasite, and the only effect of neutering was on the occurrence of ascarid infection [27]. The molecular study of cats examined revealed the presence of *T. cati* DNA, and the overall infection rate was 23% (23/100), which is relatively compared with the prevalence encountered in a disagreement with another study in Switzerland, which recorded 31.5%. *T. cati* was identified by PCR, and in Isfahan city, it was recorded at 17.7% [28,29].

According to a different study, 40% of *T. cati* cases were identified by PCR analysis using the mitochondrial DNA (mt) NADH dehydrogenase subunit 1 gene (nad1). Variations among various research results may arise from differences in the results when addressing the terms prevalence, size or number of samples, sampling methodologies, nature of samples as coprological or necropsy, other epidemiological variables, statistical analysis, the use of different PCR techniques, and different DNA extraction techniques. Furthermore, DNA extraction procedures to extract *T. cati* genomic DNA include crushing, grinding, boiling, freeze-thawing, and application of kits [30]. The findings disagreed with those of another study, which indicated 30% were from the Himalayan breed and 32% were from the Shirazi breed. It is also important to remember that a variety of conditions may impede the parasite’s eggs from being discharged in their typical form. These include sporadic excretion in the presence of evident infections and a protracted time during which the eggs do not go through embryonic development [4].

In this study, 10 PCR products had 100% similarity to the sequences of the ITS-2 region of the ribosomal DNA of *T. cati* data available in GenBank. In another study, various mitochondrial and nuclear genes were applied using a DNA-based method to amplify ITS-2, a valid region to distinguish *Toxascaris leonina* from *T. cati* [31]. Species differences within Iranian helminths isolates of *T. cati, T. canis,* and *T. leonina* were roughly 2.0%, 1.7%, and 2.6% for *pnad1*, 2.3%, 1.3%, and 1.0% for the *pcox1* gene [32].

## Conclusion

Cats are significant clinical reservoirs for zoonotic parasites. In Iraq, Baghdad has a high incidence of *T. cati* detections. Compared to conventional methods, PCR is thought to be a more sensitive, accurate diagnostic procedure that confirms species identity.

## List of Abbreviations

µl, microliter; ANOVA, analysis of variance; BLAST, basic local alignment search tool; bp, Base pair; DNA, deoxyribonucleic acid; ELISA, enzyme-linked immunosorbent assay; ITS2, internal transcribed spacer 2; LSD, least significant differences; MUC-120, mucin; NaCl, Sodium chloride; NCBI, National Center for Biotechnology Information; ng/µl, nanograms per microliter; PCR, polymerase chain reaction; RNA, ribonucleic acid; *T. cati*,* Toxocara cati*; UV, ultraviolet light; V, voltage.
